# Recent advances in the stereoselective synthesis of distal biaxially chiral molecules

**DOI:** 10.3762/bjoc.22.34

**Published:** 2026-03-16

**Authors:** Fanxing Zhou, Chen Zhang, Lingyu Sun, Yiyun Fang, Siming Zheng, Lina Hu, Mengyang Shen, Zhen Zhao, Wei Xu, Yunqiang Sun, Zi-Qiang Rong

**Affiliations:** 1 Frontiers Science Center for Flexible Electronics (FSCFE), Shaanxi Institute of Flexible Electronics (SIFE) & Institute of Flexible Electronics (IFE), Northwestern Polytechnical University (NPU), 127 West Youyi Road, Xi'an 710072, China; 2 School of Chemistry and Chemical Engineering, Linyi University, Linyi 276000, China; 3 School of Materials Science and Engineering, Linyi University, Linyi, 276000, China

**Keywords:** axially chiral compounds, stereoselective synthesis

## Abstract

Molecules bearing 1,3-dual axial and more distal axial chiralities are widely applied in chiral ligands, natural products, and anticancer agents, with their unique spatial configurations endowing them with distinctive functions and values. Although significant progress has been made in the asymmetric synthesis of distal biaxial chirality, overcoming the challenges of steric complexity and dynamic stability to achieve efficient and general construction remains a critical issue. This review summarizes recent advances in the field of distal biaxial chirality, highlighting three major synthetic strategies: direct one-step construction of distal biaxial chirality, multistep sequential generation, and conversion from central to biaxial chirality, with the aim of providing new perspectives and methodologies for further development in this area.

## Introduction

**Figure 1:**
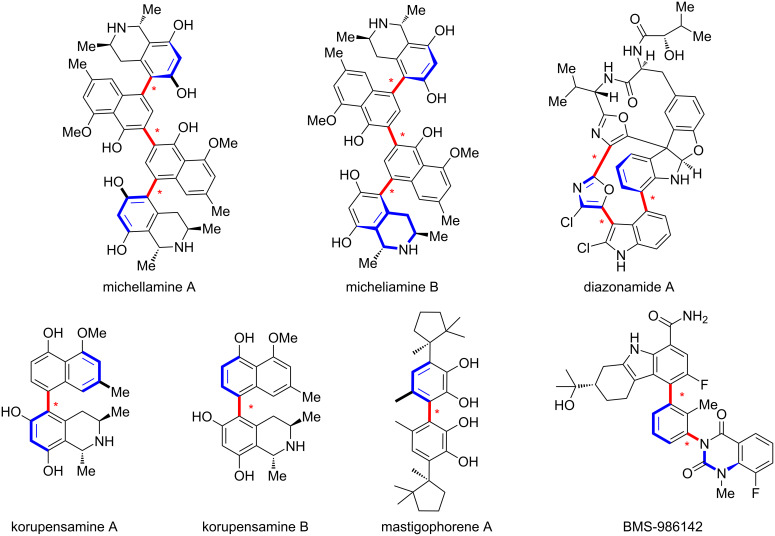
Natural products with various stereogenic axes.

In recent years, axially chiral scaffolds, which arise from hindered rotation between two planes connected by a single bond, have attracted increasing attention due to their widespread applications in chiral ligands, organocatalysts [1], and functional materials [2], making them highly valuable molecular frameworks in organic chemistry [3–24]. Axially chiral molecules form stable spatial configurations due to restricted rotation, providing a well-defined chiral environment that is advantageous in molecular recognition [25], stereochemical induction and catalysis [26]. These molecules are widely found in natural products [27] and drugs, such as michellamines A and B [28], korupensamines A and B [29], diazonamide A [30], mastigophorene A [31], and the recently developed drug candidate BMS-986142 [32] (Figure 1). When a molecule contains two or more chiral axes, it constitutes a bi- or multiaxial chiral system, significantly increasing structural complexity and stereochemical diversity, which endows the molecules with unique functionalities and excellent performance in catalysis, pharmaceuticals [33], and materials science [34].

In 1989, Hayashi, Hayashizaki, and Ito reported a highly stereoselective asymmetric cross-coupling reaction of 2-methylnaphthylmagnesium bromide with bromonaphthalene, catalyzed by a nickel complex with ferrocenylphosphine as the ligand, successfully synthesizing biaxially chiral molecules, namely 1,1':5',1"- and 1,1':4',1"-ternaphthalene compounds [35]. This work significantly advanced the development of bi- and multiaxial chirality. The synthesis of multiaxial chiral molecules requires appropriate steric hindrance to elevate the rotational energy barrier of each chiral axis [36], and the spatial interactions between axes are often difficult to control [37], particularly for remote biaxial systems (1,3-biaxial and beyond), where diastereoselectivity and enantioselectivity are challenging to regulate. Various multiaxial chiral natural products with high biological activity exist in nature [28], and in artificial synthesis, the medical field is actively exploring the potential applications of multiaxial chirality in drugs and therapeutics. The development of BMS-986142 [32] has further advanced treatment strategies for tumors and lymphocytic leukemia.

To date, the development of bi- and multiaxial chiral architectures has been partially summarized in existing reviews [38–39]. This article aims to provide a comprehensive overview of reported methods for the synthesis of remote biaxial chiral molecules, focusing on three main synthetic strategies: direct one-step synthesis of biaxial systems, sequential formation of one chiral axis followed by the second, and transformation from central chirality to axial chirality. We anticipate that this review will facilitate the development of novel synthetic strategies for remote biaxial chiral molecules, improve asymmetric synthesis efficiency, and expand their applications in catalysis, drug discovery, and functional materials.

## Review

### One-step construction of remote biaxial chiral molecules

In recent years, the development of asymmetric catalytic methods, including both transition-metal catalysis and organocatalysis, has provided powerful tools for the one-step construction of remote biaxial chiral molecules. These strategies enable precise control over the stereochemistry of multiple axes in a single reaction, allowing efficient formation of distal biaxial chiral scaffolds while maintaining excellent enantio- and diastereoselectivity. Recent studies have demonstrated that such one-step catalytic approaches not only streamline the synthesis of structurally complex biaxial molecules but also open new avenues for their applications in catalysis, drug discovery, and functional materials.

**Scheme 1:**
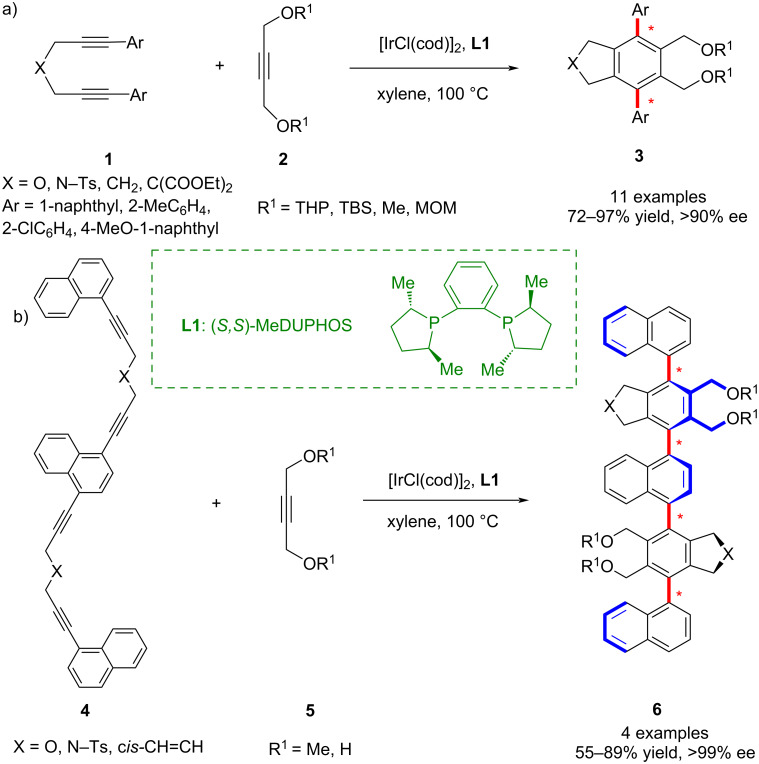
Iridium complex-catalyzed asymmetrical synthesis of axially chiral (a) teraryl compounds **3** [40] and (b) pentaaryl derivatives **6** with four consecutive chiral axes [41].

In 2004, Shibata and co-workers reported a novel iridium-catalyzed asymmetric [2 + 2 + 2] cycloaddition reaction between α,ω-diynes **1** and monoalkynes **2**, providing an alternative to traditional asymmetric coupling strategies for the synthesis of *C*_2_-symmetric biaryl chiral compounds (Scheme 1a) [40]. They further demonstrated the feasibility of constructing N-, O-, and C-containing five-membered rings with iridium complexes and examined the influence of different alcohol protecting groups on the monoalkyne substrates. The reaction proceeded in generally high yields (>70%) with satisfactory enantioselectivity.

Building on this work, Shibata and Tsuchikama developed a one-pot, transition-metal-catalyzed approach for the synthesis of helically chiral polyaryl compounds with well-defined sequences of axial chirality. Using this method, they successfully obtained pentaaryl **6** (Scheme 1b) and nonaryl derivatives (not shown) bearing four and eight consecutive chiral axes, respectively [41]. This strategy delivered excellent enantioselectivity, diastereoselectivity, and overall efficiency, underscoring its unique advantages in the synthesis of multiaxially chiral scaffolds.

**Scheme 2:**
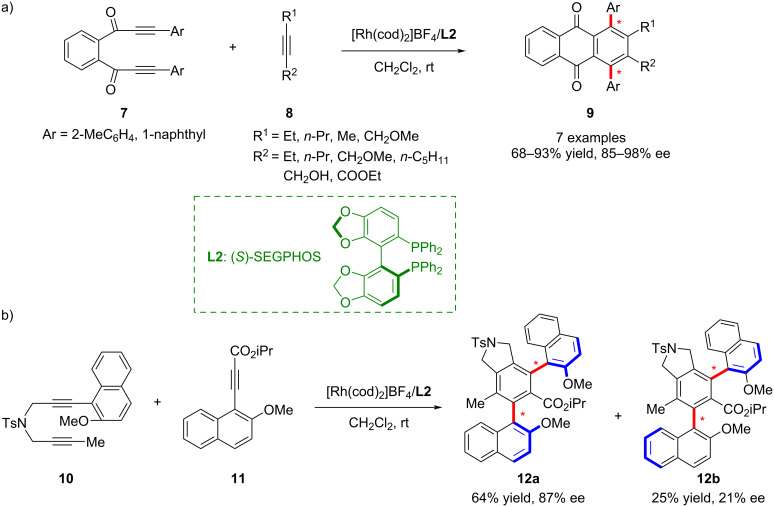
Rhodium-catalyzed enantio- and diastereoselective cycloaddition of 1,2-bis(arylpropiolyl)benzenes with monoalkynes.

In parallel, Tanaka and co-workers advanced a [2 + 2 + 2] cycloaddition strategy to realize an asymmetric transformation catalyzed by a cationic rhodium complex under ambient conditions (Scheme 2a) [42]. This protocol, involving 1,2-bis(arylpropiolyl)benzenes **7** and a monoalkynes **8**, furnished axially chiral 1,4-triaryl compounds **9** with an anthraquinone framework in excellent yield, enantioselectivity, and diastereoselectivity. Notably, this system overcame the limitations of previous methodologies that required elevated temperatures and suffered from poor efficiency and narrow substrate scope. Later, in 2011, the group extended this approach using a bisphosphine-ligated rhodium complex to efficiently synthesize carboxylic acid derivatives (Scheme 2b) [43]. They revealed that *ortho*-alkoxy substitution on the benzene-derived alkyne markedly enhanced both reactivity and enantioselectivity, while incorporation of a naphthyl substituent into the diyne enabled access to remote biaxially chiral molecules.

**Scheme 3:**
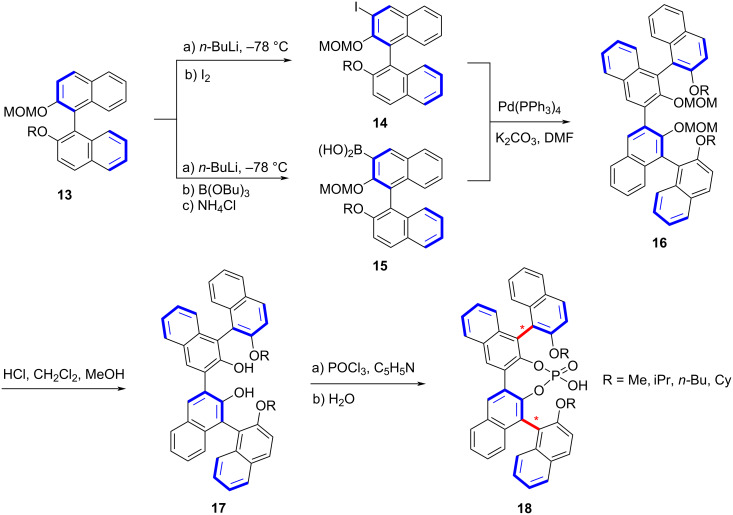
Synthesis of remote double axially chiral phosphoric acids.

Subsequent studies have further broadened the synthetic toolbox for remote biaxial chirality. Du and co-workers designed a series of chiral phosphoric acid catalysts derived from protected axially chiral diols **13**, employing a boronic acid-mediated coupling strategy to construct remote biaxially chiral phosphoric ligands (Scheme 3) [44]. The resulting catalysts **18** demonstrated remarkable efficiency in the asymmetric transfer hydrogenation of quinoline derivatives.

**Scheme 4:**
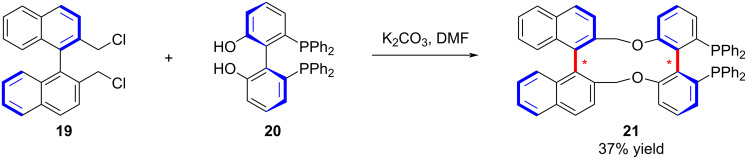
Construction of chiral biaxial diphosphine ligand.

Along similar lines, Zhang and co-workers introduced an additional axial chirality element into a ligand framework, affording a pair of diastereomeric remote biaxial chiral ligands **21** (Scheme 4) [45]. These ligands were successfully applied in asymmetric hydrogenation, revealing dual asymmetric induction effects and enriching the design principles for chiral catalytic systems.

**Scheme 5:**
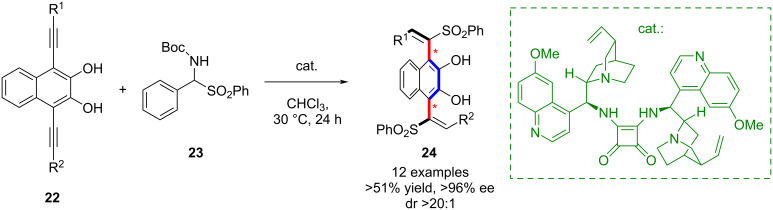
Atroposelective synthesis of biaxially chiral 1,4-distyryl-2,3-naphthalene diols.

In addition, Yan’s group developed an organocatalytic strategy for the synthesis of 1,4-divinyl compounds **24** containing a 2,3-diol motif via a vinylogous quinone methide (VQM) intermediate (Scheme 5) [46]. The transformation exhibited excellent stereoselectivity, and preliminary studies showed that the products could be elaborated into novel axially chiral scaffolds with potential applications in ligand and catalyst development.

**Scheme 6:**
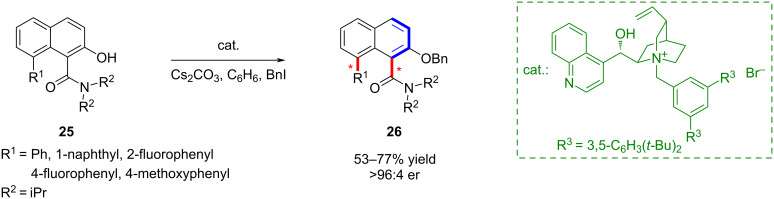
H-Bond-enabled enantioselective synthesis of remote biaxially chiral amides mediated by the counterion.

In a complementary direction, Smith’s group achieved the highly enantioselective synthesis of axially chiral naphthamides (Scheme 6) [47]. Their strategy employed transition-state hydrogen bonding to induce substrate deracemization, followed by alkylation for dynamic kinetic resolution. Moreover, additional alkylation at a sterically congested second rotational axis enabled the construction of remote, double axially chiral molecules **26**.

**Scheme 7:**
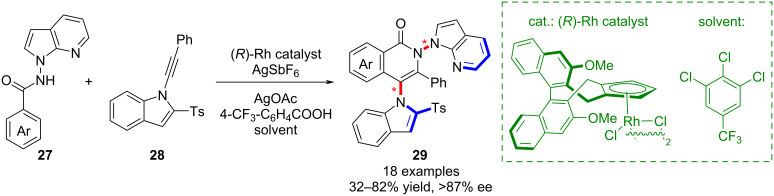
Enantioselective synthesis of biaryl products with twofold chiral axes.

Further advances have come from transition-metal catalysis. Li’s group established a rhodium-catalyzed protocol for the synthesis of remote biaryl scaffolds bearing both N–N and N–C chiral axes, using benzamides **27** and alkynylindoles **28** as substrates (Scheme 7) [48]. During an extensive investigation of reaction conditions and substrate scope they also were able to synthesize various diaxially chiral N–N and C–C derivatives through reaction of 1-alkynylnaphthalenes with benzamides. In this context they observed that the stereoselectivity of the alkyne insertion could be tuned by solvent effects, particularly with hexafluoroisopropanol, which induced inversion of the C–C axial configuration (not shown).

**Scheme 8:**
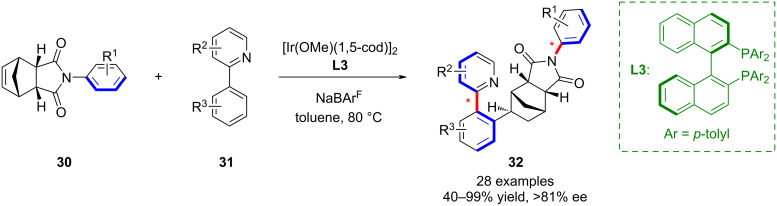
Iridium-catalyzed C–H alkylation to obtain the distal biaxial atropisomers.

In a related development, our group reported an iridium-catalyzed asymmetric alkylation for the efficient construction of distal biaxial molecules **32** incorporating both C–C and C–N chiral axes (Scheme 8) [49]. The method delivered atropisomers in high yield and stereoselectivity. Moreover, investigation of the photophysical properties revealed that the enantiomers exhibited promising features for chiral functional materials, including circularly polarized luminescence (CPL).

**Scheme 9:**
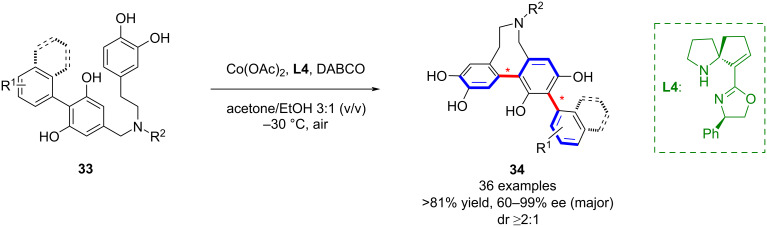
Co/SPDO-catalyzed biaxial bridged terphenyl compounds.

More recently, Tu’s group described a cobalt-catalyzed direct oxidative coupling of phenols, enabling the synthesis of axially chiral bridged teraryl molecules **34** (Scheme 9) [50]. This method provided access to biaxial bridged eight-membered terphenyl atropisomers with broad substrate scope, high yields, and operational simplicity under air.

**Scheme 10:**
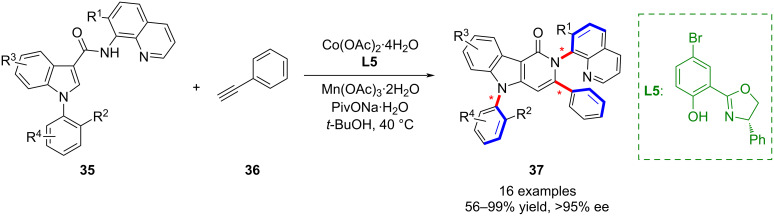
Atroposelective Co-catalyzed synthesis of pyridoindolones with two distinct C–N axes.

In parallel, Shi’s group developed a cobalt-catalyzed asymmetric synthesis of remote C–N biaxial chiral pyridoindolones **37**, affording enantioenriched products with distinct chiral axes (Scheme 10) [51]. The reaction displayed a broad substrate scope (up to 60 examples), excellent enantioselectivity and diastereoselectivity, and the products exhibited high photoluminescence quantum yields, indicating potential utility in chiral fluorescent materials.

**Scheme 11:**
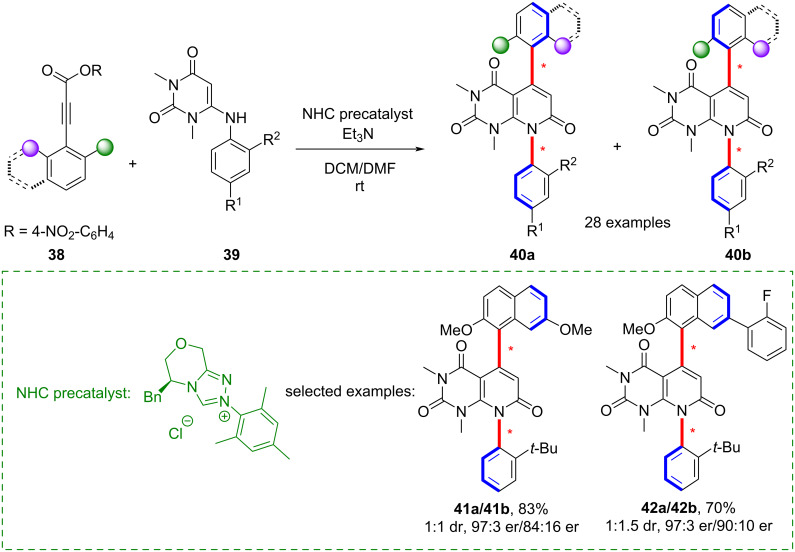
NHC organocatalytic synthesis of fused 1,4-biaxial uracils with C–C and C–N chiral axes.

In addition, Du’s group reported an N-heterocyclic carbene (NHC)-catalyzed (3 + 3) cycloaddition of 2,6-disubstituted alkyne esters **38** with 6-aminouracils **39**, affording distal biaxial uracil frameworks with both C–C and C–N chiral axes (Scheme 11) [52]. A preliminary biological evaluation revealed inhibitory activity of selected products against MV4-11 cancer cells, highlighting their potential in pharmaceutical research. At the same time, efficient synthetic routes to uracils bearing a single C–N axis were also achieved in yields exceeding 60%.

### Sequential construction of distal biaxial chirality via stepwise axis formation

Although one-step catalytic methods enable the efficient and direct construction of remote biaxially chiral molecules, the spatial interactions between multiple chiral axes can make it challenging to fully control the stereochemistry in a single transformation. This has motivated the development of sequential strategies, in which one chiral axis is constructed first, followed by the introduction of the second axis in a subsequent step. Such stepwise approaches allow precise control over each axis, improving the stereoselectivity of remote biaxial chiral molecules and providing greater flexibility in the design and synthesis of complex multiaxial systems. Building on the one-step strategies discussed above, sequential formation of chiral axes has thus emerged as another important methodology for the synthesis of remote biaxially chiral compounds.

**Scheme 12:**
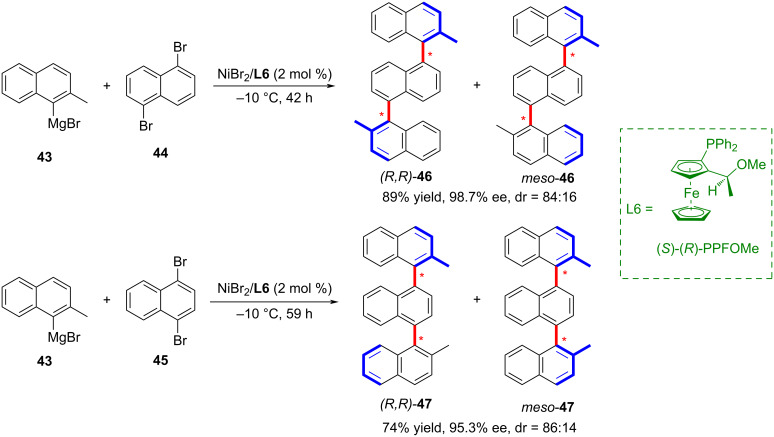
Synthesis of the first biaxially chiral compound reported by Ito and co-workers [35].

In 1989, Ito and co-workers reported the synthesis of the first distally biaxially chiral compound (Scheme 12) [35]. This reaction employed nickel as transition-metal catalyst, for the cross-coupling of 2-methyl-1-naphthylmagnesium bromide (**43**) with 1,5- (**44**) and 1,4-dibromonaphthalenes (**45**). Notably, the reaction achieved the synthesis of ternaphthalenes with excellent optical purity exceeding 95% ee.

**Scheme 13:**
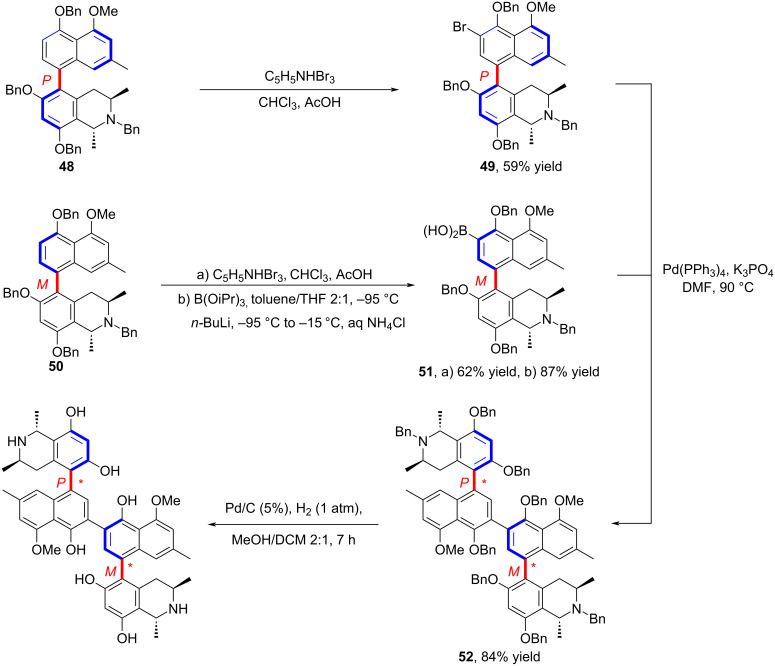
Synthesis of chiral homoaryl compounds by Suzuki–Miyaura coupling.

In 2014, Tang and co-workers pioneered the first asymmetric synthesis of functionalized chiral biaryl natural products with high enantioselectivity via Suzuki–Miyaura coupling (Scheme 13) [29]. The reactions were carried out under mild conditions and required only low catalyst loading. The key to achieving high enantioselectivity lies in the stereoselective synthesis of the starting compounds which relies on polar–π interactions between an aryl component containing a highly polarized BOP bis(2-oxo-3-oxazolidinyl)phosphonic substituent and the extended π system in the arylboronic acid coupling partner.

**Scheme 14:**
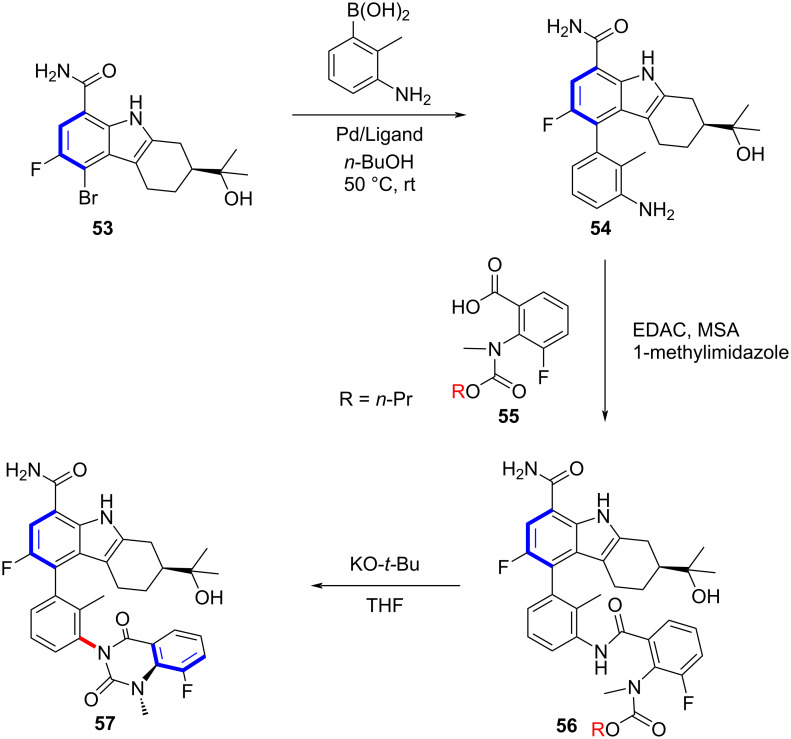
Structurally complex APIs with multiple chiral axes.

In 2018, Razler and co-workers described an approach for the synthesis of an architecturally complex API **57** having multiple chiral axes (Scheme 14) [53]. Their strategy relied on prioritizing the construction of axially chiral diastereomers with the highest energy barriers to reduce the venture of subsequent epimerization. At the same time, the higher barrier diastereomer served as a chiral template for building the stereoconfiguration of axial chiral bonds. Remarkably, this strategy has explored a broader space for the pharmaceutical industry, enabling the highly efficient synthesis of structurally complex compounds.

**Scheme 15:**
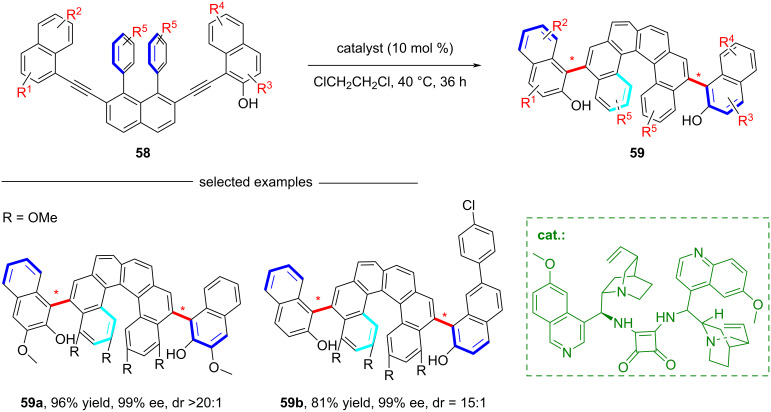
Synthesis of helicenes containing stereogenic axes.

In 2019, Yan and co-workers achieved the efficient asymmetric synthesis of chiral helicenes **59** with biaxial chirality via a vinylidene *ortho*-quinone methide (VQM) intermediate, which undergoes intramolecular nucleophilic cyclization (Scheme 15) [54]. This work represents the first synthesis of a compound integrating both helical and axial stereogenic elements, and achieves high diastereoselectivity and enantioselectivity through a cinchona alkaloid-derived squaramide organocatalytic approach. The mechanistic study revealed that the reaction proceeds through a stepwise double cyclization process: The first cyclization generates an intermediate bearing a stereogenic axis, while the second cyclization involves dynamic kinetic resolution of the spiral reaction intermediate under catalyst control, ultimately forming a helix and another stereogenic axis.

**Scheme 16:**
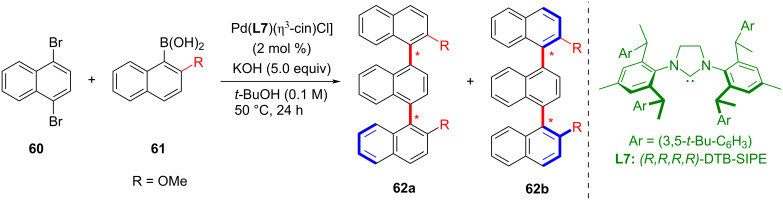
Chiral NHC–Pd complex-catalyzed Suzuki–Miyaura cross-coupling reaction for the synthesis of block-transfer isomeric biaryl compounds.

In 2019, Shi and co-workers developed a Suzuki–Miyaura cross-coupling reaction catalyzed by a chiral NHC–Pd complex proceeding with high enantioselectivity (Scheme 16) [55]. This reaction can be used to synthesize different kinds of atropisomeric biaryls and heterobiaryls and is also applicable to the construction of tetra-*ortho*-substituted biaryls. This represents the first example of a C(sp^2^)–C(sp^2^) cross-coupling reaction catalyzed by a chiral NHC–metal complex.

**Scheme 17:**
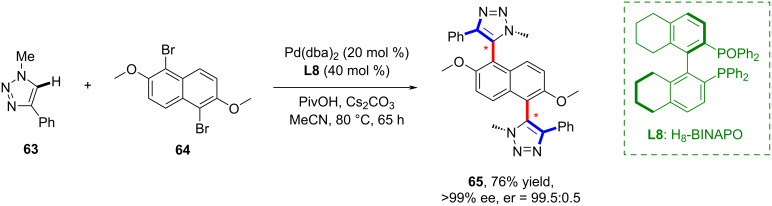
Highly enantioselective C–H arylation of heteroarenes.

In 2020, Baudoin and co-workers reported an approach to synthesize atropisomeric (hetero)biaryls (Scheme 17) [56]. This highly enantioselective C–H arylation of heteroarenes employs a Pd(0) complex with H_8_-BINAPO **L8** as the chiral ligand, enabling the arylation of 1,2,3-triazoles and pyrazoles in excellent yields with great selectivity. This method also facilitates stereoselective diarylation, allowing the construction of two stereogenic axes.

**Scheme 18:**
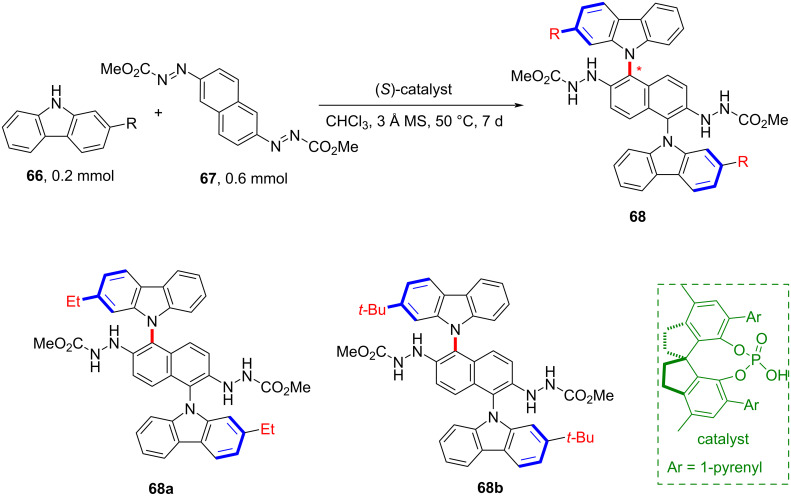
Synthesis of novel axially chiral N-arylcarbazole skeletons by the assembly of azidonaphthalenes and carbazoles through chiral phosphoric acid catalysis.

In 2020, Tan and co-workers pioneered an organocatalytic strategy for assembling azo-naphthalenes with carbazoles to construct novel chiral N-arylcarbazole frameworks **68** (Scheme 18) [57]. Additionally, this work also represents the first enantioselective C–H amination of arenes catalyzed by a chiral phosphoric acid. Thus, this nucleophilic aromatic substitution reaction not only enables the synthesis of compounds bearing two chiral N-aryl axes but also provides a viable alternative to metal-catalyzed C–N cross-coupling reactions.

**Scheme 19:**
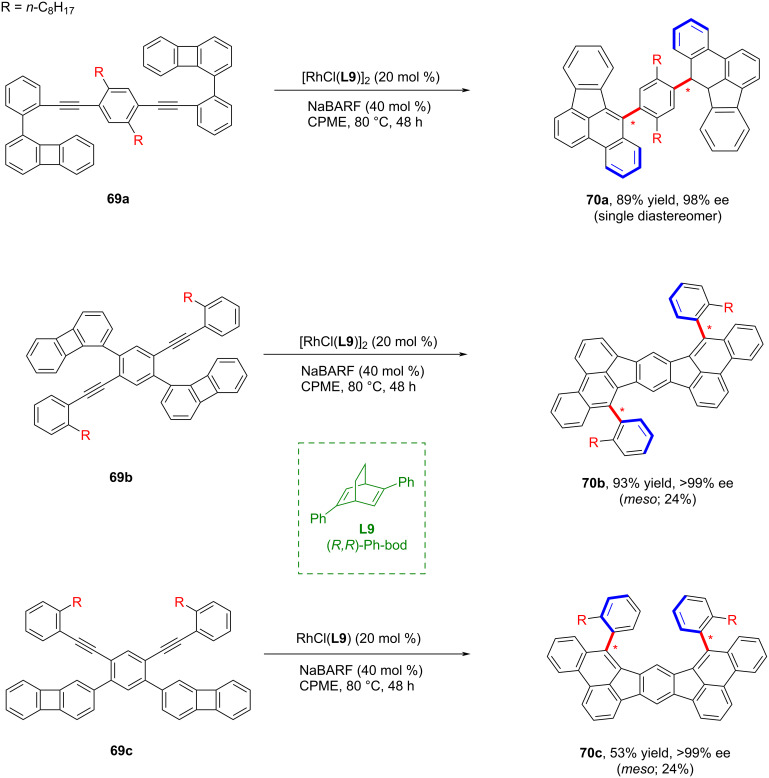
Catalytic enantioselective synthesis of axially chiral polycyclic aromatic hydrocarbons.

Soon after, Shibata and co-workers developed a cycloisomerization strategy that generated various axially chiral polycyclic aromatic hydrocarbons (PAHs) **70** through bond-cleavage followed by successive cyclization reactions with excellent yields and enantioselectivity (Scheme 19) [58].

**Scheme 20:**
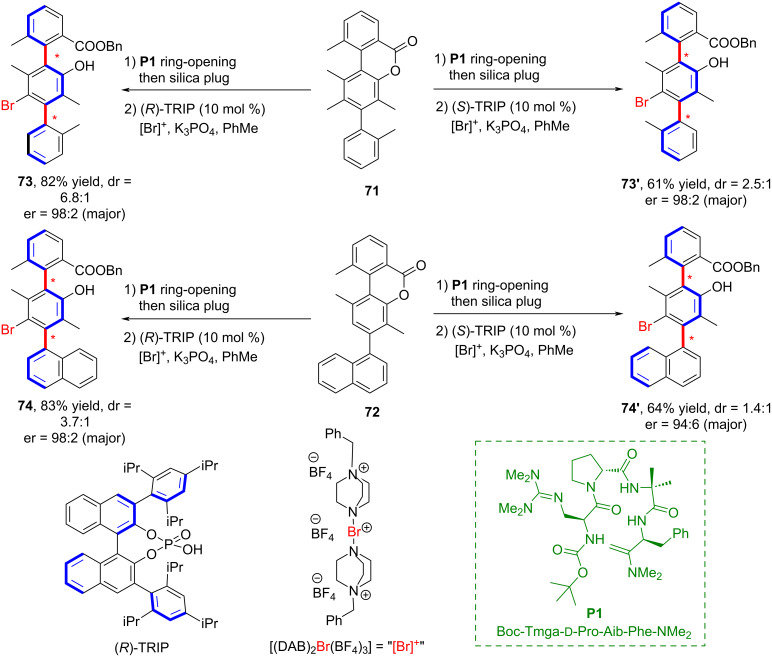
Catalytic synthesis of biaxial triphenylene block-transfer isomers.

In 2020, Miller and co-workers reported a class of Brønsted basic guanidinylated peptides that were able to catalyze atroposelective chlorination reactions, representing the first reported example of such a transformation (Scheme 20) [59]. This reaction employs a complementary strategy for the catalytic synthesis of biaxial terphenyl atropisomers with chlorinated and brominated variants to achieve high diastereo- and enantioselectivity. The process takes place via a two-step kinetic resolution.

**Scheme 21:**
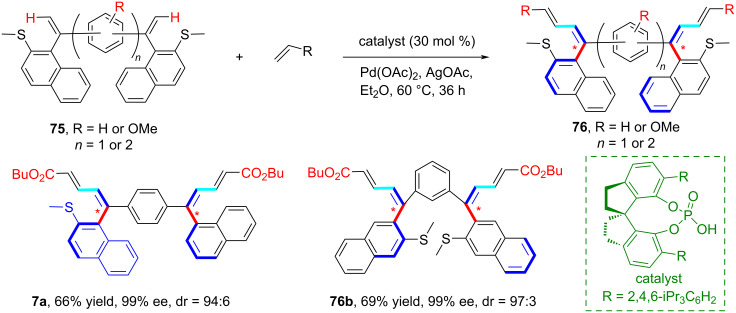
A Pd(II)-catalyzed *trans*-selective C–H alkenylation strategy through thioether-directed olefination.

In 2021, Shi and co-workers reported a highly *trans*-selective synthesis of axially chiral styrenes **76** containing a conjugated 1,3-diene scaffold though a Pd(II)-catalyzed strategy involving a thioether-directed alkenyl C–H olefination (Scheme 21) [60]. Pleasingly, the strategy also enabled the synthesis of stereoisomers having two stereogenic axes with high enantioselectivity. The axially chiral styrenes produced in this reaction may find application as novel S-olefin ligands.

**Scheme 22:**
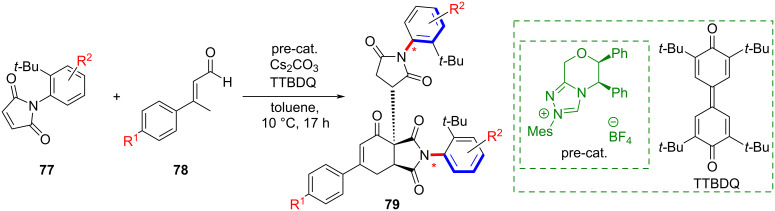
Synthesis of *N*-arylphthalimides from prochiral maleimides and NHC-activated dienolides.

In 2023, Amatore and co-workers developed an NHC-catalyzed method to acquire previously unknown axially chiral *N*-arylphthalimides **79** (Scheme 22) [61]. This system adopted a (4 + 2) oxidative annulation strategy. The products are generated through the reaction of prochiral *N*-arylmaleimides with NHC-derived chiral dienolates and contain as many as four chiral centers along with distal biaxial chirality.

**Scheme 23:**
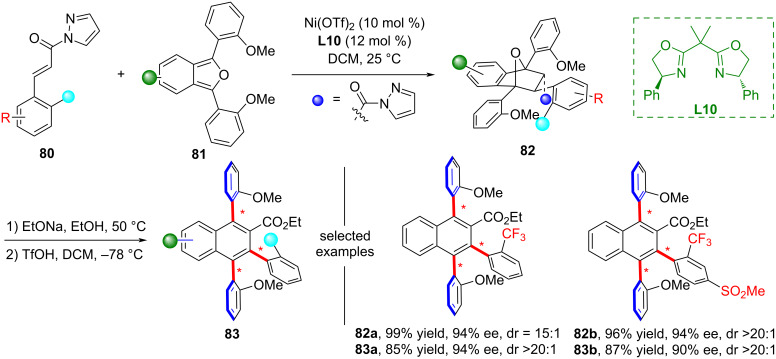
Ni-catalyzed synthesis of triaxially chiral polysubstituted naphthalene scaffolds.

In 2025, Cai and co-workers developed an efficient approach for constructing triaxially chiral polysubstituted naphthalene scaffolds **82** (Scheme 23) [62]. This method successfully employed a Ni(II)-catalyzed Diels-Alder reaction between 1,3-biaryl-isobenzofurans and α,β-unsaturated N-acyl pyrazoles, followed by a TfOH-promoted dehydration aromatization step. These atropisomeric compounds, which contain multiple stereogenic axes, show broad application prospects in the field of chiral organic material design.

**Scheme 24:**
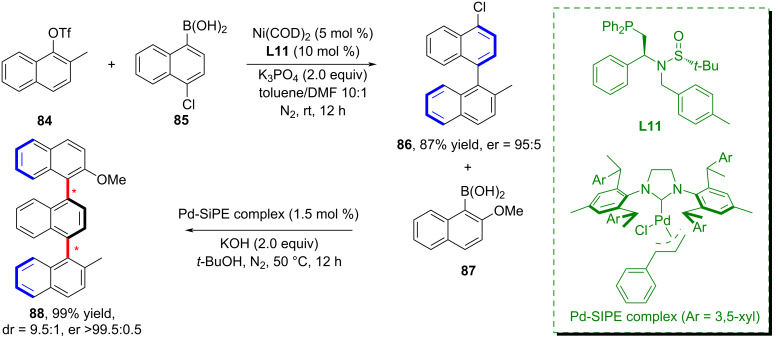
Enantioselective Ni-catalyzed Suzuki–Miyaura cross-coupling reaction.

In 2025, Hong and co-workers successfully predicted a series of novel Sadphos (sulfinamide phosphine) ligands and accomplished the first enantioselective Ni-catalyzed Suzuki–Miyaura cross-coupling reaction (Scheme 24) [63]. Their work validated the utility of machine learning in predicting the synthetic feasibility of ligands. By integrating a wide range of Pd catalysis data with limited Ni/Sadphos data through a co-modeling approach, this work pioneered a few-shot learning strategy for the design of molecular catalysts.

### Transformation from central to axial chirality for constructing distal biaxial systems

Building on the advances in the direct one-step construction of distal biaxial chiral molecules and sequential formation of each chiral axis, another powerful strategy emerged that exploits central chirality as a stereochemical template to induce axial chirality. In this approach, the initial formation of one or more stereogenic centers establishes a defined three-dimensional framework, which subsequently guides the generation of one or more chiral axes through cyclization, oxidative aromatization, or related transformations. This central-to-axial strategy not only expands the toolbox for constructing remote biaxial and multiaxial chiral systems but also offers enhanced stereocontrol, especially for sterically congested or electronically challenging substrates. As such, it serves as a complementary and highly effective route for the assembly of complex distal biaxial architectures that are difficult to access via direct or stepwise axis formation alone.

**Scheme 25:**
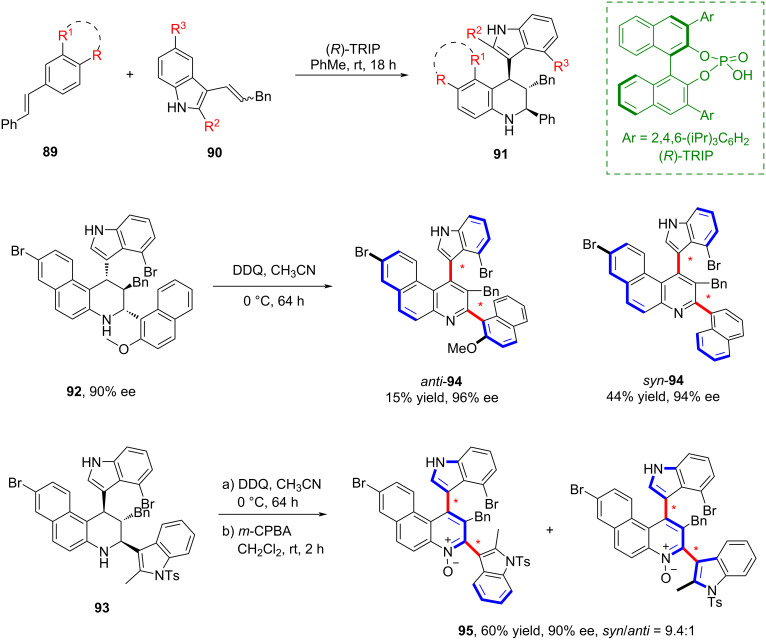
The stereoselective synthesis of axial chiral indole–quinoline systems.

In 2019, Bertuzzi and co-workers achieved the first enantioselective synthesis of an axially chiral indole–quinoline system (Scheme 25) [64]. They employed a central-to-axial oxidative strategy that proceeded with high stereoselectivity and demonstrated that the organocatalytic asymmetric Povarov cycloaddition between 3-alkenylindoles and *N*-arylimines provides an efficient approach to synthesize highly hindered tetrahydroquinolines. Notably, this method offered a novel strategy for constructing compounds bearing two stereogenic axes.

**Scheme 26:**
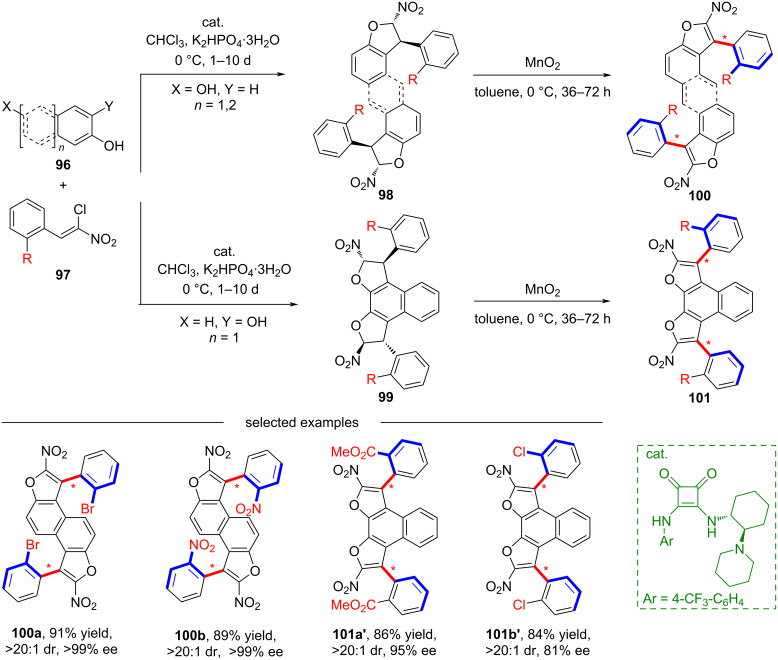
The synthesis of bisbenzofuran atropisomeric oligoarenes containing two distal C–C stereogenic axes.

In 2020, Bonne and co-workers developed a bidirectional enantioselective strategy for synthesizing bisbenzofuran atropisomeric oligoarenes containing two distal C–C stereogenic axes (Scheme 26) [65]. Using simple and readily accessible dehydroxylated aromatics **96** and chloronitroalkenes **97** as starting materials, they synthesized the key enantioenriched central chiral dihydrobenzofuran precursor through an organocatalyzed domino reaction. This unique bidirectional catalyst-controlled strategy successfully achieved the transformation from central chirality to axial chirality via oxidative aromatization.

## Conclusion

Distally biaxially chiral molecules play crucial roles in the fields of asymmetric catalysis, drug synthesis, and materials science due to their unique biaxial chirality and distal position. However, multiaxial sterical systems still commonly suffer from the challenges of difficult stereoselectivity control and low conformational stability. For this reason, numerous attempts have been made towards the efficient construction of distally biaxially chiral skeletons, and a variety of synthetic strategies such as [2 + 2 + 2] cycloaddition and desymmetrization of latent chiral molecules. In this review, we summarized the research progresses related to the asymmetric synthesis of distal biaxial systems in recent years from single-step to multistep approaches. It is anticipated that new chiral skeletons and asymmetric synthesis methods of distally biaxial systems will be developed with the progress of research in the future, thereby facilitating further breakthroughs in this area.

## Data Availability

Data sharing is not applicable as no new data was generated or analyzed in this study.
